# Association between female cardiometabolic index and infertility: a population-based study

**DOI:** 10.3389/fpubh.2025.1513358

**Published:** 2025-02-24

**Authors:** Junqian Liu, Fengya Zhu, Yuan Wang, Jie Wu

**Affiliations:** ^1^College of Acupuncture and Massage, Chengdu University of TCM, Chengdu, China; ^2^Hospital of Chengdu University of Traditional Chinese Medicine, Chengdu, China

**Keywords:** cardiometabolic index, obesity, lipid metabolism, infertility, NHANES, cross-sectional study

## Abstract

**Background:**

Visceral fat accumulation and dyslipidaemia are associated with infertility symptoms. The cardiometabolic index (CMI) is a comprehensive quantitative measure of central obesity and dyslipidaemia. However, the link between the female CMI and the couple infertility needs to be explored further. Hence, this study aimed to dissect this connection.

**Methods:**

The study used a cross-sectional approach to select 1,641 female participants from the National Health and Nutrition Examination Survey (NHANES), which was conducted between 2013 and 2020. The natural log–transformed CMI (In-CMI) was used to consider the non-normal distribution of CMI. Logistic regression models adjusted for covariates were employed to investigate the association between the In-CMI and couple infertility.

**Results:**

After adjusting for all covariates, each 1 unit increase in the In-CMI was associated with a 34% increase in the incidence of infertility (odds ratio [OR] 1.34, 95% confidence interval [CI] 1.10–1.64, *p* = 0.004). In addition, the association remained statistically significant after dividing the In-CMI into tertiles (T1, T2, and T3). The T3 group, with the highest In-CMI, showed higher odds of infertility compared with the T1 group (OR 2.11, 95% CI 1.38–3.23, *p* < 0.001). Subgroup and interaction analyses revealed that the association between the In-CMI and infertility depended on a history of treatment for pelvic infection (*P* for interaction <0.05). The inflection point for a positive linear association between In-CMI and infertility was−0.73.

**Conclusion:**

The female CMI is linked to the incidence of couple infertility. Moreover, the female CMI shows significant medical significance for assessing couple infertility risk of childbearing age.

## Background

Infertility is characterized by the inability to achieve a successful pregnancy after 12 months of regular, unprotected sexual intercourse (1). Infertility has been ranked alongside malignant tumors and cardiovascular diseases as one of the top three diseases affecting quality of life (2). The inability to have children affects men and women throughout the world and leads to pain, depression, discrimination and rejection (3, 4). The prevalence of infertility ranges from 3 to 30% (5). Therefore, it is necessary to take appropriate preventive measures to reduce the negative impact of infertility and the pressure on society.

Obesity has become one of the most severe public health issues. As of 2022, more than 1 billion people worldwide are obese. Notably, the prevalence of obesity in women is particularly alarming, especially central obesity (6). Central obesity is characterised by excessive fat accumulation around the stomach and an increase in waist circumference. Central obesity is associated with metabolic disorders, including dyslipidaemia, and infertility (7). Obesity leads to intracellular fat accumulation or elevated circulating lipids, and approximately 45% of women of childbearing age are affected by dyslipidaemia (8). In recent years, several conventional body size assessments, such as waist circumference and body mass index (BMI), have been widely used to measure the increase in obesity. However, these do not consider lipid metabolism as well as visceral and subcutaneous fat deposition (9). It has been argued that these BMI-like metrics are deceptive and ignore lipid metabolic profiles (10).

The cardiometabolic index (CMI) was first introduced by researchers as a comprehensive assessment of abdominal obesity and fat metabolism (11). Moreover, the CMI can be a valuable indicator in people with normal weight but metabolic disorders (10). The CMI may provide a more robust and independent indicator of metabolic abnormalities than traditional anthropometric measurements (12). Recently, there has been increased discussion on the relationship between the CMI and many diseases, such as depression and diabetes, suggesting its value as an indicator of metabolic disorders (13, 14). However, there has been limited exploration of the link between the CMI and infertility. The CMI may play have an as-yet unexplored clinical role in infertility. Therefore, this study explored this link using the National Health and Nutrition Examination Survey (NHANES) database.

## Methods

### Data collection

**Figure 1:**
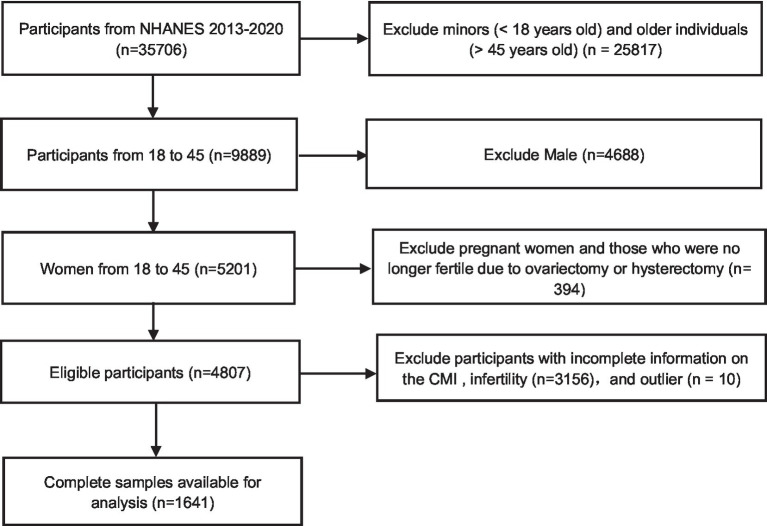
Flowchart.

In the United States, a cross-sectional survey called NHANES, conducted by the Centers for Disease Control and Prevention, assesses the health and nutrition status; it includes face-to-face interviews and physical examinations of the population. Participants first provide written informed consent and then complete a health interview at home; finally, a physical examination is conducted at a mobile medical centre where urine and blood samples are collected. The present study used data collected from 2013 to 2020 as part of the NHANES. For this period, there were 35,706 people. After excluding minors (< 18 years old) and older individuals (> 45 years old) (*n* = 25,817), men (*n* = 4,688), pregnant women and those who were no longer fertile due to ovariectomy or hysterectomy (*n* = 394) and individuals with incomplete information on the CMI (*n* = 2,387) or infertility (*n* = 769), and outlier (*n* = 10). A total of 1,641 eligible subjects were analysed (Figure 1).

### CMI

The CMI was available for the 1,641 eligible subjects. It was calculated as follows:


$$\begin{array}{l} CMI=\left[\mathrm{waist circumference}\;\left(\mathrm{cm}\right)\times TG\;\left(\mathrm{mmol}/L\right)\right]\\ \;/\left[\mathrm{height}\;\left(\mathrm{cm}\right)\times HDL-C\;\left(\mathrm{mmol}/L\right)\right] \end{array}$$

Where TG is the triglyceride level and HDL-C is the high-density lipoprotein cholesterol level, both measured in the blood.

### Assessment of infertility

Infertility was assessed based on the responses to two questions of the NHANES: (1) “Have you tried to get pregnant for at least 1 year without getting pregnant?” (2) “Have you ever visited a doctor or other health care provider because you could not get pregnant?” Answers of “refused” or “do not know” to either question were considered as missing information on infertility and were excluded. Women who answered “yes” to either of these questions were considered to have a history of infertility. The remaining participants had no history of infertility.

### Covariates

Various sociodemographic indicators, lifestyle habits and factors related to fertility were considered to be potential confounding factors that could affect the study results. The sociodemographic factors included age, race/ethnicity, education level, marital status and the poverty-to-income ratio (PIR). Lifestyle factors including smoking/alcohol consumption status and physical activity. There were four levels for the drinking status: no drinking, moderate drinking (no more than one drink per day), heavy drinking (1–4 drinks per day), and binge drinking (at least four drinks per day) (15). In the U.S., adults are recommended to perform at least 75 min of high-intensity exercise or 150 min of moderate-intensity physical activity per week to promote health. The 1,641 eligible subjects were divided into three categories according to their physical activity: active, moderate and low. Other covariates included height, age at menarche, use of birth control pills, pregnancy history, and treatment for pelvic infections or pelvic infection drug/PID. Besides, the missing data for categorical variables were coded using missing indicator categories, including marital status (*n* = 171), education (*n* = 171), alcohol consumption status (*n* = 119), ever taken birth control pills (*n* = 723), and pregnancy history (*n* = 171). Missing data were subsequently handled using multiple imputation methods.

### Statistical analysis

The continuous demographic data are presented as the mean and standard deviation or median and upper and lower quartiles; the categorical data are described as a number depending on the infertility status. Given that the CMI showed a skewed distribution, a natural logarithmic transformation was used to adjust the data to a normal distribution (the In-CMI). Participants in the 2 groups (group with history of infertility, group without history of infertility) were separately divided into three groups (T1, T2, T3) according to ln-CMI (T1, T2, and T3); T1, with the lowest In-CMI, was set as the benchmark group. The chi-square test and *t*-test were used to evaluate the socioeconomic information of the subjects based on the In-CMI. Multiple and logistic regression was used to explore the relationship between the In-CMI and infertility. In the model 1, there were no modifications based on the covariates. The model 2 was adjusted for age and race. The model 3 was adjusted for age, race, the PIR, age at menarche, education level, smoking behavior, drinking habits, reproductive history, marital status, use of contraceptives, treatment of pelvic infections/PID, and frequency of physical activity. After converting the In-CMI from continuous to categorical data (tertiles), trend analysis was used to explore whether there was a linear relationship between the In-CMI and infertility, including subgroup analysis of age groups, age at menarche, smoking habits, drinking habits, pregnancy history, contraceptive use and PID treatment history. The nonlinear relationship between infertility and In-CMI was studied. The correlation between infertility and In-CMI was analysed by employing smoothed curve fitting. The threshold effect of each interval was calculated and fitted using a piecewise regression model. R Studio (version 4.2.2) and Empower Stats (version 4.0) were used for statistical analysis. A *p* < 0.05 was considered to be statistically significant.

## Results

### Population characteristics

This study identified 1,641 women aged between 18 and 45 years who took part in the NHANES study, including 197 with infertility. The mean age of all included women was 31.11 ± 8.27 years, while the mean age of the women with infertility was 34.15 ± 7.07 years. Table 1 provides specific information on the infertility status of the participants. Infertility was more common in women who were older, married or cohabiting, better educated, had a history of alcohol abuse, were current smokers, had a history of pregnancy, had been treated for pelvic infections/PID and had been taking birth control pills. The In-CMI was significantly higher for the women with infertility compared with the women without infertility. We divided the women into three groups based on the In-CMI: T1 (≤ 0.222), T2 (0.222–0.466) and T3 (> 0.466). The rate of infertility increased significantly as the In-CMI increased: 20.81% for T1, 35.53% for T2 and 43.66% for T3 (*p* < 0.001).

**Table 1:** Characteristics of the study participants: NHANES 2013–2020.

		No history of infertility	History of infertility	*p*-value
Characteristics	Overall	1,444	197	
Age, mean ± SD (years)	31.11 ± 8.27	30.69 ± 8.33	34.15 ± 7.07	<0.001
Ratio of family income to poverty	2.25 ± 1.54	2.22 ± 1.53	2.43 ± 1.64	0.11
Age when first menstrual period occurred	12.45 ± 1.76	12.48 ± 1.76	12.25 ± 1.77	0.098
Race, *n* (%)				0.348
Non-Hispanic white	280 (17.06)	249 (17.24)	31 (15.74)	
Non-Hispanic Black	163 (9.93)	149 (10.32)	14 (7.11)	
Mexican American	525 (31.99)	451 (31.23)	74 (37.56)	
Other Hispanic	376 (22.91)	334 (23.13)	42 (21.32)	
Other multiracial	297 (18.10)	261 (18.08)	36 (18.27)	
Marital status, *n* (%)				<0.0001
Married or living with partner	855 (52.10)	709 (49.10)	146 (74.11)	
Widowed/Divorced/separated	361 (22.00)	333 (23.06)	28 (14.21)	
Single/never married	254 (15.48)	234 (16.21)	20 (10.15)	
Education, *n* (%)				<0.0001
Less than high school	232 (14.14)	199 (13.78)	33 (16.75)	
High school	280 (17.06)	247 (17.11)	33 (16.75)	
More than high school	958 (58.38)	830 (57.48)	128 (64.98)	
Alcohol consumption status, *n* (%)				<0.0001
None	306 (18.65)	287 (19.88)	19 (9.65)	
Moderate	391 (23.83)	340 (23.55)	51 (25.89)	
Heavy	642 (39.12)	577 (39.96)	65 (33.00)	
Binge	183 (11.15)	141 (9.76)	42 (21.32)	
Smoking status, *n* (%)				0.042
Never smoker	1,203 (73.31)	1,073 (74.31)	130 (65.99)	
Former smoker	180 (10.97)	151 (10.46)	29 (14.72)	
Current smoker	258 (15.72)	220 (15.24)	38 (19.29)	
Ever taken birth control pills, *n* (%)				0.076
Yes	598 (36.44)	513 (35.53)	85 (43.15)	
No	320 (19.50)	290 (20.08)	30 (15.23)	
Pregnancy history, *n* (%)				<0.0001
Has never been pregnant before	423 (25.78)	386 (26.73)	37 (18.78)	
Has been pregnant before	1,047 (63.80)	890 (61.63)	157 (79.70)	
Ever treated for a pelvic infection/PID, *n* (%)				0.003
Yes	69 (4.21)	53 (3.67)	16 (8.12)	
No	1,572 (95.80)	1,391 (96.33)	181 (91.88)	
Physical activity level, *n* (%)				0.989
Inactive	900 (54.85)	791 (54.78)	109 (55.33)	
Less active	126 (7.68)	111 (7.69)	15 (7.61)	
active	615 (37.48)	542 (37.54)	73 (37.06)	
CMI	0.33 (0.19–0.57)	0.32 (0.03–4.27)	0.41 (0.07–8.24)	<0.001
CMI group, *n* (%)				<0.001
T1 group	547 (33.33)	506 (35.04)	41 (20.81)	
T2 group	547 (33.33)	477 (33.03)	70 (35.53)	
T3 group	547 (33.33)	461 (31.93)	86 (43.66)	

Data was shown as number (percentage), mean ± SD or median (25th–75th percentile). SD, Standard Deviation; CMI, cardiometabolic index; T, team.

### Relationship between infertility and the CMI

**Figure 2:**
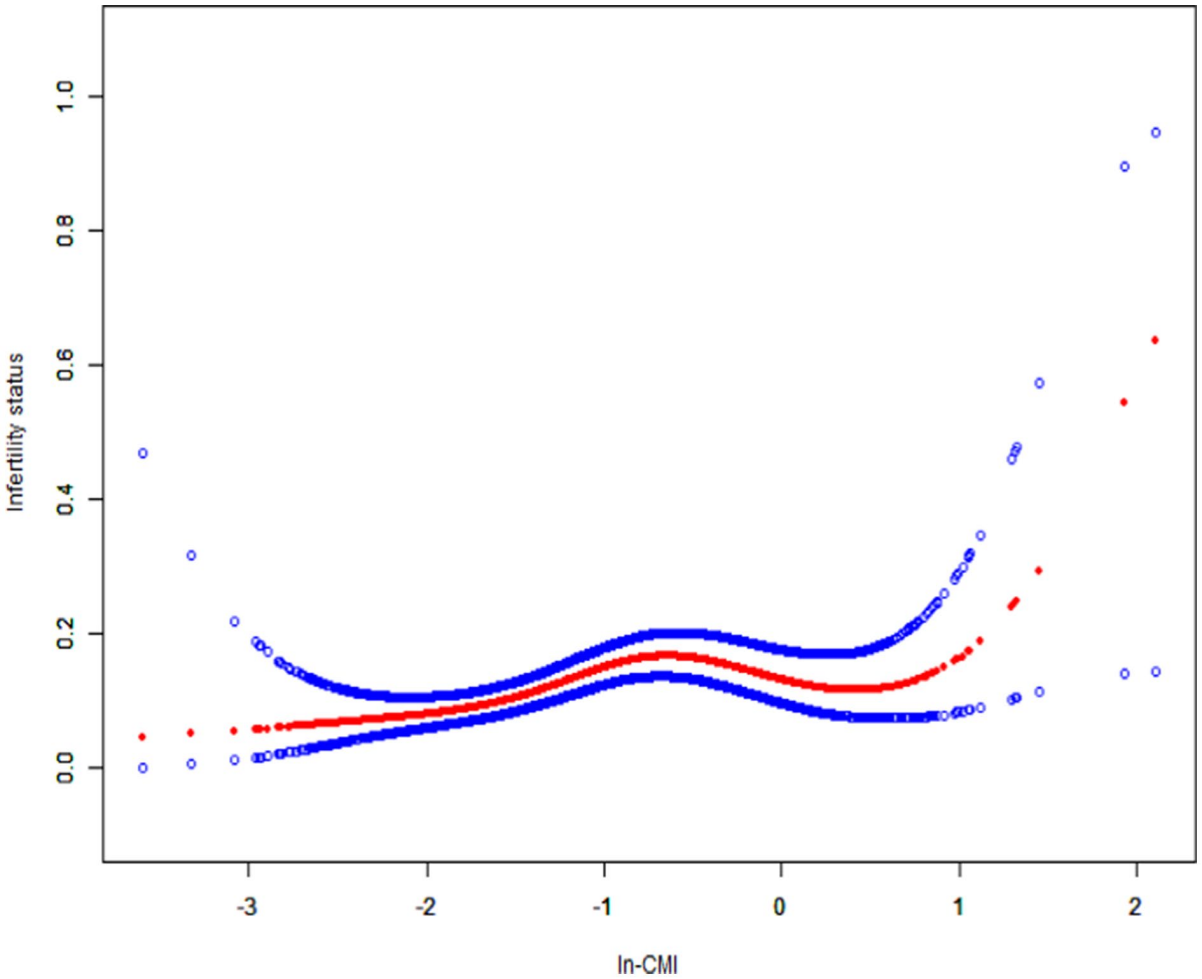
Smoothed curve fitting for In-CMI and infertility. The solid red line represents the smooth curve fit between variables. Blue bands represent the 95% confidence interval from the fit. In-CMI, Natural log-transformed cardiometabolic index.

A higher In-CMI was associated with a higher risk of difficulty conceiving. All models indicated a positive association between infertility and the In-CMI. Multivariate logistic regression analysis in the model 1 revealed that the odds of infertility increased by 41% for every 1-unit increase in the In-CMI (odds ratio [OR] 1.41, 95% confidence interval [CI] 1.18–1.68, *p* < 0.001). When considering the three groups based on the IN-CMI, the T2 and T3 groups were more likely to have difficulty conceiving children than the T1 group (T2: OR 1.81, 95% CI 1.21–2.72, *p* = 0.005; T3: OR 2.30, 95% CI 1.55–3.41, *p* < 0.001). In the model 3, the probability of infertility increased by 34% when the In-CMI increased by 1 unit (OR 1.34, 95% CI 1.10–1.64, *p* = 0.004). Besides, the T2 and T3 groups were associated with increased odds of infertility (T2: OR 1.78, 95% CI 1.17–2.72, *p* = 0.007; T3: OR 2.11, 95% CI 1.38–3.23, *p* < 0.001) (Table 2). A smooth curve fitting analysis was conducted to explore the relationship between ln-CMI and couple infertility. A standard linear model indicated a positive association between ln-CMI and infertility risk (OR = 1.34, 95% CI: 1.10–1.64, *p* = 0.004). However, a two-piecewise linear regression model revealed a turning point at ln-CMI = −0.73. For ln-CMI < −0.73, the OR was 1.89 (95% CI: 1.31–2.71, *p* = 0.001), whereas for ln-CMI ≥ −0.73, the association was not significant (OR = 0.87, 95% CI: 0.56–1.34, *p* = 0.518) (Figure 2 and Table 3).

**Table 2:** A greater In-CMI is linked to a higher risk of infertility.

Variable	Model 1		Model 2		Model 3	
	OR (95% CI)	*P*-value	OR (95% CI)	*P*-value	OR (95% CI)	*P*-value
Continuous variables						
In-CMI	1.41 (1.18, 1.68)	<0.001	1.31 (1.09, 1.58)	0.005	1.34 (1.10, 1.64)	0.004
Categorical variable						
T1 Group	Ref		Ref		Ref	
T2 Group	1.81 (1.21, 2.72)	0.004	1.79 (1.16, 2.63)	0.007	1.78 (1.17, 2.72)	0.007
T3 Group	2.30 (1.55, 3.41)	<0.001	2.04 (1.36, 3.06)	<0.001	2.11 (1.38, 3.23)	<0.001
p for trend		<0.001		<0.001		<0.001

Insensitivity analysis, the cardiometabolic index was converted from a continuous variable to a categorical variable (tertiles). 95% CI, 95% confidence interval; OR, Odds ratio; In-CMI, Natural log-transformed cardiometabolic index; T, Team; Ref, Reference; Model 1, No covariates were adjusted; Model 2, Adjusted for age and race; Model 3, Adjusted for age, race, PIR, age when first menstrual period occurred, education, smoking status, alcohol consumption status, pregnancy history, marital status, birth control pills and pelvic infection/PID, physical activity level.

**Table 3:** Analysis of the threshold effect of In-CMI on the prevalence of infertility.

Threshold effect analysis of ln-CMI on infertility a two-piecewise linear regression model.	
Fitting by standard linear model	1.34 (1.10, 1.64) 0.004
Fitting by two-piecewise linear model	
Breakpoint (K)	-0.73
In-CMI < −0.73	1.89 (1.31, 2.71) 0.001
In-CMI ≥ − 0.73	0.87 (0.56, 1.34) 0.518
P for log-likelihood ratio	0.020

The results of the threshold effect of In-CMI on the prevalence of infertility was adjusted for age, race, PIR, age when first menstrual period occurred, education, smoking status, alcohol consumption status, pregnancy history, marital status, birth control pills and pelvic infection/PID, physical activity level.

### Subgroup analysis

Subgroup analyses showed that after stratifying patients based on a history of treatment for pelvic infection/PID, the In-CMI was positively associated with the incidence of infertility in women with pelvic infection (OR 5.36, 95% CI 1.39–20.75, *p* = 0.015) and women without pelvic infection (OR 1.32, 95% CI 1.07–1.62, *p* = 0.009). The association between the In-CMI and infertility depended on a history of treatment for pelvic infection (*P* for interaction <0.05). However, the association between the In-CMI and infertility did not depend on age, age at menarche, the smoking status, alcohol use, pregnancy history, use of birth control pills and BMI (Table 4).

**Table 4:** Subgroup analysis.

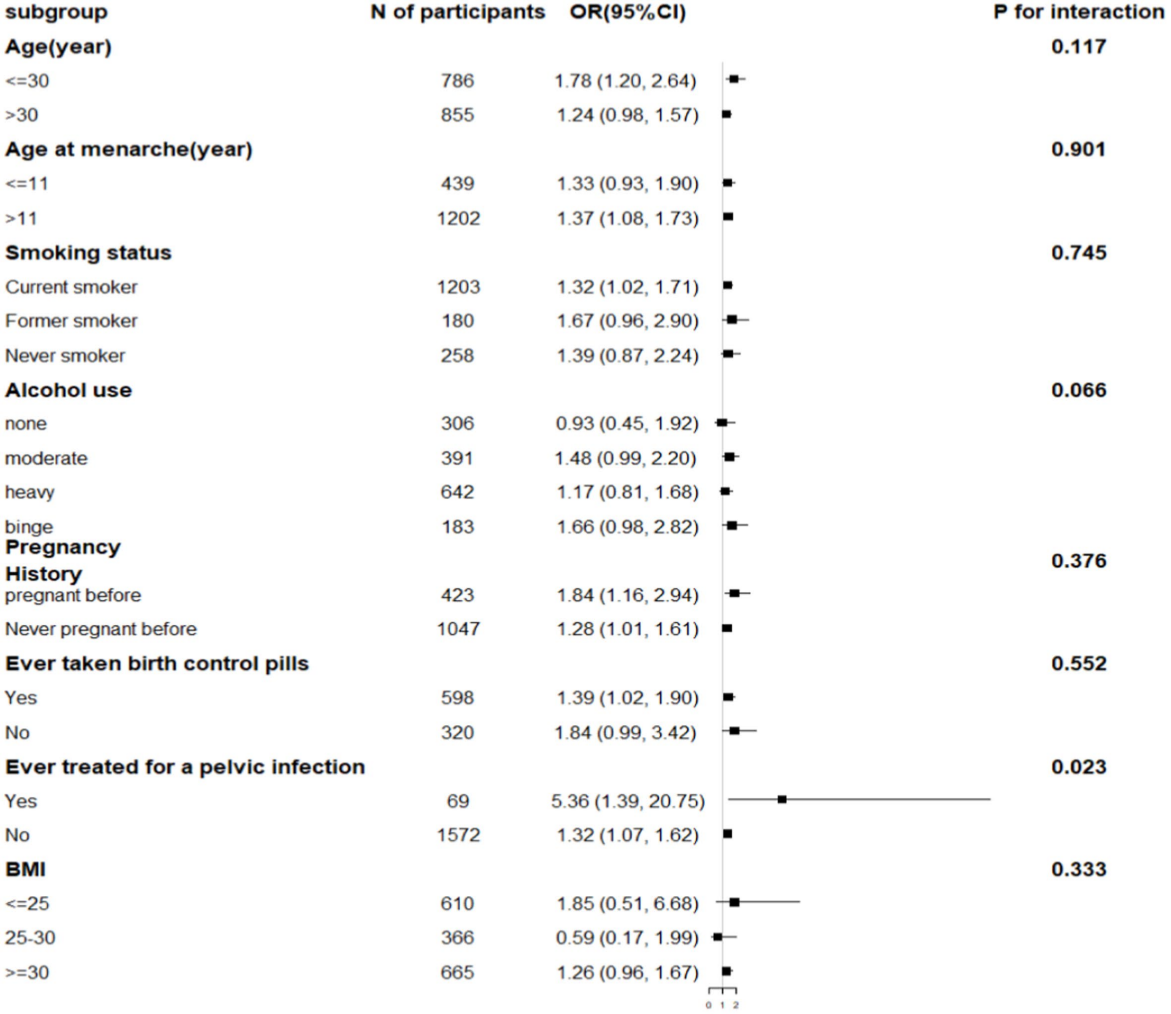

N, number; OR, odds ratio; 95% CI, 95% confidence interval; BMI, body mass index.

### Receiver operating characteristic (ROC) curves of CMI, BMI in relation to infertility

To evaluate the efficacy of BMI and CMI in predicting infertility, we constructed receiver operating characteristic (ROC) curves. The ROC curve was a graphical tool for assessing the performance of classification models by plotting the true positive rate (degree of sensitivity) and the false positive rate (degree of specificity) across various threshold settings. The area under the curve (AUC) served as a crucial metric for gaging the predictive performance of the model, with an AUC value closer to 1 indicating better predictive performance. In this study, we computed the ROC curves for BMI and CMI, and obtained their respective AUC values. The AUC for BMI was 0.598 (95% CI 0.56–0.64), while the AUC for CMI was 0.585 (95% CI 0.55–0.62) (Supplementary Figure 1).

## Discussion

In this cross-sectional study of 1,641 subjects, it found that a higher In-CMI is associated with a higher incidence of infertility. Subgroup and interaction analyses revealed that the association between the In-CMI and infertility depended on a history of treatment for pelvic infection/PID. This research used a piecewise regression model and found a positive correlation when the In-CMI was < −0.73. When the In-CMI was > −0.73, the association with infertility was no longer statistically significant.

This study is the first to assess the relationship between couple infertility and the female CMI. The CMI is an indicator of centripetal obesity, reflecting the distribution of obesity and lipid metabolism. Abdominal obesity, characterised by visceral fat, is closely related to metabolic disorders and is a significant risk factor for infertility (16). Visceral fat is a crucial indicator of obesity and lipid metabolism (17), and its distribution also significantly affects hormone concentrations. Zhang et al. (18) conducted a retrospective study of 10,424 patients with infertility divided into two groups – one with normal lipid metabolism and one with dyslipidaemia – and found that dyslipidaemia had a negative impact on pregnancy outcomes for *in vitro* fertilization of patients with infertility but not polycystic ovary syndrome. A study by Tortoriello et al. (19) showed that diet induced a 60% decrease in spontaneous pregnancy rate in obese mice. According to previous studies, abnormalities in lipid cycle metabolism affect the hormonal environment (20), ovarian and uterine function (21), which in turn may contribute to infertility. A randomized, double-blind trial found that reduced fertility was associated with abnormal lipid levels in women (8).

In this exploratory study of 1,641 adult women, it showed that a higher In-CMI may increase the probability of infertility. In model 3, each 1-unit increase in In-CMI was associated with a 34% increase in the probability of infertility after adjusting for age, race, the PIR, age at menarche, education level, smoking status, alcohol consumption status, pregnancy history, marital status, use of birth control pills, a history of treatment for pelvic infection/PID and the physical activity level. Moreover, the T2 and T3 groups with the highest In-CMI had increased odds of infertility compared with the T1 group. In addition, this study validated the association between the In-CMI and infertility based on subgroup analyses. The association depended on a history of treatment for pelvic infection/PID. On the one hand, visceral adipose tissue functions as an active endocrine organ, secreting inflammatory factors such as tumor necrosis factor-alpha (TNF-*α*) and interleukin-6 (IL-6). The excessive release of these factors can lead to chronic inflammatory responses, causing damage to vascular endothelial cells and thereby increasing the risk of developing chronic pelvic inflammatory conditions (22, 23). On the other hand, Obesity and PID involve distinct pathological mechanisms but share a common feature of inflammatory responses. For instance, the chronic low-grade inflammation associated with obesity may impact reproductive health through systemic inflammatory pathways, while PID induces localized inflammation that can lead to tubal scarring and dysfunction. Despite these differing mechanisms, both conditions may contribute to infertility through inflammation-related processes.

The mechanisms that underlie the strong association between the female CMI and couple infertility are unclear, but lipid metabolism may be responsible for these results. For example, phospholipids, as a member of lipids, are also an important component of cell membranes. It is involved in membrane fluidity and signaling, thus affecting the process of ovulation by follicular rupture (24, 25). Lipids, on the other hand, as an important source of energy, also provide energy support for cellular functions including follicular rupture (26). Thus lipid metabolism may affect processes such as ovulation and pregnancy. Previous studies have found that gene expression of 11β-hydroxysteroid dehydrogenase type1 (11β-HSD1) is positively associated with abdominal obesity (27). 11β-HSD1 is considered to be a determinant of local glucocorticoid function, which can be activated by site-specific overexpression and activation of local glucocorticoids. Excess glucocorticoids can malignantly cause visceral adipose tissue accumulation on the one hand and insulin resistance on the other. Insulin resistance leads to increased ovarian testosterone secretion, decreased secretion of sex hormone-binding globulin, and changes in adipokine levels, which disrupts the balance of the hypothalamic–pituitary-ovarian axis, leading to infertility (28). In women with central obesity, excessive abdominal lipid accumulation leads to the breakdown of metabolites such as fatty acids and prostaglandins, which increases ovarian steroid hormone secretion (29). This process predisposes the body to chronic inflammation (30) and localized endometrial autophagy (31). Which affect the process of embryo development during reproduction and lead to infertility (32, 33). Additionally, changes in steroid hormone secretion are closely related to aging. As women age, their fertility declines significantly, accompanied by a decrease in metabolic rate, alterations in fat distribution, and lipid metabolism dysregulation. These changes may affect reproductive health through various mechanisms.

BMI, as a simple and easily accessible anthropometric indicator, facilitates large-scale screening and monitoring, and offers certain reference value in assessing obesity levels and infertility risks. However, BMI solely considers the relationship between weight and height, failing to comprehensively reflect an individual’s metabolic status. It cannot distinguish between the weight of adipose tissue and other types of tissue. Therefore, in some circumstances, BMI may misestimate an individual’s infertility risk. In contrast, CMI integrates multiple metabolic parameters, enabling a more comprehensive assessment of an individual’s degree of metabolic abnormality and thus a more accurate prediction of infertility risk. Despite the slightly lower AUC of CMI compared to BMI in this study, we believe that CMI still holds great potential in predicting infertility. Firstly, CMI requires the results of multiple biochemical indicators and may be influenced by factors such as sample size and measurement errors in certain situations. Secondly, by incorporating multiple metabolic parameters, CMI theoretically provides a more comprehensive reflection of an individual’s metabolic abnormalities, allowing for a more precise prediction of infertility risk. Additionally, CMI may offer unique predictive advantages in specific populations, such as those with polycystic ovary syndrome, which can contribute to a deeper understanding of the pathogenesis of infertility. Due to database limitations, further validation is not possible at this stage, necessitating future research to explore this area further.

### Study strengths and limitations

In summary, we comprehensively evaluated the relationship between the CMI and infertility and confirmed the predictive value of the CMI for infertility, especially when there is a history of pelvic infection. The optimal critical value of In-CMI for predicting infertility was −0.73. Specific lipidomic changes often predict the onset of clinical symptoms, so the CMI can provide more in-depth and cutting-edge insights for women of reproductive age. The CMI has the advantage of being cost-effective and easily measurable and provides insights into lipid metabolism. The results of this study provide potential new perspectives on infertility treatment and help to deepen the understanding of women’s reproductive health.

However, given the limitations of the study, further in-depth studies and multi-centre validation are still needed to ensure that these findings can be applied more widely in clinical practice. First, the cross-sectional nature of this study precludes the ability to establish a causal relationship between the CMI and infertility. Second, despite considering multiple covariates, it could not rule out all potential confounding influences. Additionally, the measurement of CMI in this study was based on data collected at the time of testing, whereas infertility history reflects a retrospective report of past conditions. This time gap introduces a potential limitation in accurately linking current CMI values with past infertility events, as metabolic changes or other factors influencing fertility may have occurred over time. Therefore, while CMI can provide insights into the current metabolic status, its direct correlation with infertility history may not fully account for all temporal and physiological changes that may have occurred since the infertility event. Lastly, our study has a limitation that reproductive disorders, which may potentially impact fertility as covariates, were not considered in the analysis. This limitation is primarily attributed to the lack of detailed data on common fertility-related conditions (such as endometriosis and polycystic ovary syndrome) in the NHANES database from 2013 to 2020.

## Conclusion

The female CMI is linked to the incidence of couple infertility. Moreover, the female CMI shows significant medical significance for assessing couple infertility risk of childbearing age.

## Data Availability

The original contributions presented in the study are included in the article/Supplementary material, further inquiries can be directed to the corresponding authors.
