# Supplementary material for: Association between female cardiometabolic index and infertility: a population-based study
Source: Front Public Health. 2025 Feb 24;13:1513358. doi: 10.3389/fpubh.2025.1513358 (PMC11891880; doi:10.3389/fpubh.2025.1513358)
Supplement: Supplementary file 1 [file Supplementary_file_1.docx]

#### Supplementary Figure 1. ROC curves of CMI、BMI in relation to infertility


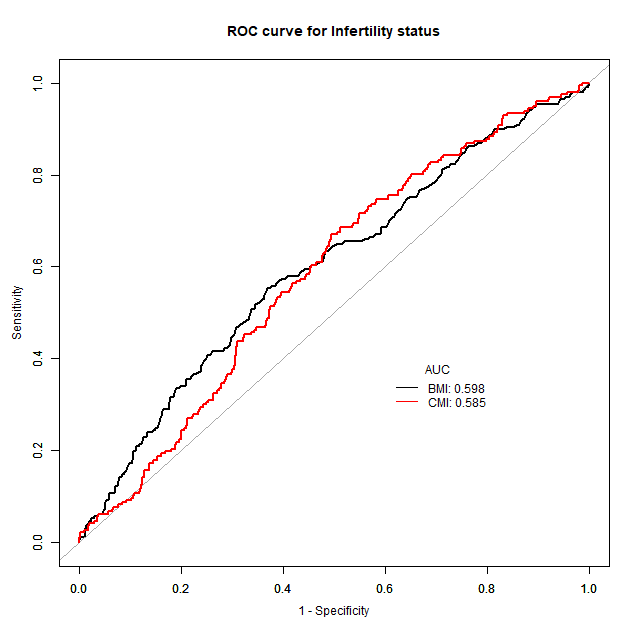


Abbreviations: ROC, Receiver operating characteristic; AUC, Area under the curve; BMI, body mass index; CMI, cardiometabolic index.
